# Gene trees and species trees: irreconcilable differences

**DOI:** 10.1186/1471-2105-13-S19-S15

**Published:** 2012-12-19

**Authors:** Krister M Swenson, Nadia El-Mabrouk

**Affiliations:** 1 Département d'Informatique (DIRO), Université de Montréal, H3C 3J7, Canada; 2 McGill Centre for Bioinformatics, McGill University, H3C 2B4, Canada

## Abstract

**Background:**

Reconciliation is the classical method for inferring a duplication and loss history from a set of extant genes. It is based upon the notion of embedding the gene tree into the species tree, the incongruence between the two indicating evidence for duplication and loss. However, results obtained by this method are highly dependent upon the considered species and gene trees. Thus, painstaking attention has been given to the development of methods for reconstructing accurate gene trees.

**Results:**

This paper highlights the fact that errors in gene trees are not the only reasons for the inference of an erroneous duplication-loss history. More precisely, we prove that, under certain reasonable hypotheses based on the widely accepted link between function and sequence constraints, even a well-supported gene tree yield a reconciliation that does not correspond to the true history. We then provide the theoretical underpinnings for a conservative approach to infer histories given such gene trees. We apply our method to the mammalian interleukin-1 (IL) gene tree, that has been used as a model example to illustrate the role of reconciliation.

## Introduction

### Background

Duplication followed by modification is a major mechanism driving evolution. A significant obstacle obscuring our understanding of this mechanism is the inference of duplication and loss histories for a gene family. In 1979 Goodman et al. [1] introduced gene tree and species tree reconciliation as a method to infer such a history, along with the implied orthology and paralogy relationships between the genes. A typical reconciliation study first constructs a gene family by identifying genes among a set of genomes that share certain sequence similarity [2]. Such genes are assumed to be homologs (*i.e*. originating from a single ancestral gene). A gene tree that best reflects the evolution of the sequences is then constructed. A reconciliation consists of "embedding" this gene tree into the species tree and interpreting the incongruence between the two as a description of gene family evolution through duplication and loss. As there can be several reconciliations for a given tree pair, a natural approach is to select one reconciliation, or a subset of reconciliations, that optimize some probabilistic [3-5] or combinatorial [6] criterion such as the number of duplications (*duplication cost*), of losses (*loss cost*) or of both (*mutation cost*).

If the gene tree represents the true evolutionary relationship on the gene set, then a reconciliation-based method is likely to give an accurate duplication and loss history. However uncertainty on gene trees is a serious limitation to reconciliation. In particular, it has been reported that a few misplaced leaves in the gene tree can lead to a completely different history, possibly with significantly more duplications and losses [2,7,8]. Thus, a great deal of effort has been put into finding accurate gene trees in the presence of variable rates of evolution and in the presence of horizontal gene transfer [9-18]. Other issues associated with erroneous gene trees have been addressed from a practical point of view by manually curating some orthologs (TreeFam [19]), by manually correcting gene trees (PANTHER [20]), or by avoiding reconciliation by integrating the orthology identification procedure with the gene tree construction procedure [21].

However, even when the gene tree is statistically well-supported by the data, the reconciliation approach can lead to an erroneous duplication and loss history for the gene family. The reason is that the gene tree that best reflects the sequence similarity of the gene copies is not necessarily the true phylogeny for the gene family. In particular, homologous gene copies that are responsible for preserving an original ancestral function are likely to diverge at a lower rate than the copies that are not constrained by function. Such copies are therefore likely to appear as a subtree of the gene tree, even though they are not the most evolutionary closely related copies. The link between function and sequence constraint has been largely accepted and reported in the literature [22], although no formal study has yet been conducted as the effect of such link on reconciliation.

### Results

In this paper, we formally study the consequence of functional constraints on reconciliation. Our main result (Corollary 1) is a proof that there are certain simple conditions under which reconciliation fails, even when the gene tree is perfectly well supported (*e*.*g*. every edge has high bootstrap support) and there is no horizontal gene transfer. Formally, under the hypothesis that at least one subtree of the gene tree respects the "isolocalization property" -- a property based on the link between function and sequence -- we show that reconciliation will never yield a true history if such history has a duplication event descending from a speciation event.

This not only supports the need for efforts to efficiently find accurate gene trees and compute histories through probabilistic methods, but also raises the question, "what does the relationship between the gene and species tree tell us when the gene tree respects the isolocalization property?" We provide some foundational theory on the subject; we pose two fundamental problems associated with this question and provide an algorithm to solve the simpler of the two, under the duplication cost. Finally, we apply our methods to the mammalian interleukin-1 (IL) gene tree.

In the next two sections we introduce concepts related to reconciliation and distinguish between the various types of gene homology using the terminology recommended by Fitch [23].

## Preliminary concepts and notation

### Trees

**Figure 1:**
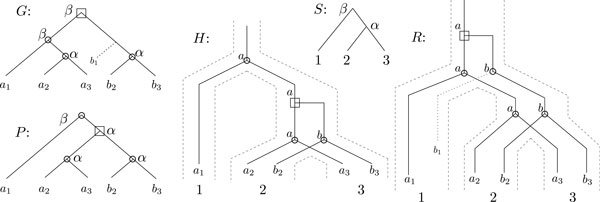
**S is a species tree for Σ = {1, 2, 3}; H represents a history, consistent with (*i.e*. embedded in) the species tree *S*, with one duplication event preceding the speciation event leading to genomes 2 and 3**. Speciation events appear as bifurcations at obtuse angles, while duplication events appear at right angles. We represent the information on isorthology by positioning the retainer of parental function directly under the parental gene. Moreover, we label isorthologs with the same letter (all *a*'s are pairwise isorthologous, and all *b*'s are pairwise isorthologous); **P **is the phylogeny for the gene family Γ = {*a*_1_,*a*_2_,*a*_3_,*b*_2_,*b*_3_} corresponding to *H*; it is the same tree as *H*, embedded differently (uncross edges). **G **is the gene tree respecting the isolocalization property that is likely to be obtained for the gene family Γ that evolved according to *H*. Internal node labels of *S*,*G*, and *P *correspond to the LCA mapping, and squares mark duplication nodes, and circles mark speciations resulting from the mapping. **R **is the reconciliation corresponding to the mapping. The loss has a dotted line indicating the lost lineage.

For our purposes, a genome is just a collection of genes. A *phylogeny *is a rooted binary tree, uniquely leaf-labeled by some set. A *species tree S *is a phylogeny over a set of species ∑ , which represents the evolutionary relationship between these species. Similarly, we can consider the evolutionary relationship between a family of genes Γ, that appear in the genomes of ∑ : a *gene tree G *for Γ is a phylogeny accompanied by a function *g *: Γ→∑ indicating the species where each gene is found. It is reasonable to assume that there is at least one gene per species in *S*. In Figure 1, the tree *S *is the species tree for ∑={1,2,3}, and *G *and *P *are two possible gene trees for Γ = {*a*_1_, *a*_2_, *a*_3_, *b*_2_, *b*_3_}, where *s*(*x_i_*) = *i *for *x *∈ {*a*, *b*}.

Given a tree *T *and a node *x *of *T*, we denote by *T_x _*the subtree of *T *rooted at *x *(*i.e*. the tree comprised of all the nodes descending from *x*). $L\left(T_{x}\right)$ is the set of leaves of *T_x _*and $S\left(T_{x}\right)$ the subset of Σ corresponding to $L\left(T_{x}\right)$$\left(i.e\;S\left(T_{x}\right)=\left\{s\left(l\right):l\in L\left(T_{x}\right)\right\}\right)$. The *lowest common ancestor *(LCA) of leaves *x *and *y *in a tree *T*, written *lca_T _*(*x*, *y*), is the common ancestor of *x *and *y *that is farthest from the root. For simplicity, we will not distinguish between the node *lca_T _*(*x*, *y*), and the ancestral gene copy present at this node. The LCA of a set of leaves is the highest LCA (closest to the root) over every pair of leaves in the set.

### Histories

We study the evolution of a family of genes taken from genomes ∑  through duplications and losses. Formally, a *loss *is an operation that removes one gene copy from an extant or ancestral genome, while a *duplication *is an operation that copies a *parent *gene to a new location in a genome. After a duplication, we call the parent gene the *source *and the copied gene the *sink *of the duplication.

Given a set of genes Γ and a function g:Γ→∑ indicating the species where each gene is found, a *duplication/loss/speciation history *(simply called a *history *in the rest of this paper) is a tree reflecting the evolution of the set from a single ancestral copy through duplication, loss and speciation events. Informally, a history has multiple subtrees of a species tree *S *appearing in it. It is said to be *consistent *with *S*. In Figure 1, *H *and *R *are two possible histories for Γ = {*a*_1_, *a*_2_, *a*_3_, *b*_2_, *b*_3_}. Speciation events appear as bifurcations at obtuse angles, while duplication events appear at right angles. Losses are represented by dotted edges; the leaf labeled *b*_1 _is a loss. Both histories *H *and *R *in Figure 1 are consistent with the species tree *S*. A formal definition of "consistent" can be found in [24].

As the true gene tree is unknown, phylogenetic information is usually inferred from molecular data. In this paper, we will distinguish between a *phylogeny for a gene family *Γ which reflects the true evolution of the gene family, and the *gene tree *for Γ, which is a tree obtained from the observed gene sequences (*e.g*. a multiple alignment of the sequences, the observed gene positions, or any other footprint of evolution observed in the extant species). In Figure 1,*G *is the gene tree for Γ = {*a*_1_, *a*_2_, *a*_3_, *b*_2_, *b*_3_}, whereas *P *is the true phylogeny of Γ corresponding to history *H*.

### Reconciliation

A *reconciliation *is a history that can be obtained from a gene tree by inserting loss leaves and labeling internal nodes as speciation or duplication nodes such that the history is consistent with the species tree. In the history *R *(which is a reconciliation of *S *and *G*) from Figure 1, the loss of *b*_1 _is implied by the duplication at the root, and the fact that there is only a single gene mapped to genome 1. Refer to [24-26] for a more detailed definition.

The parsimony criteria used to choose among the large set of possible reconciliations are the number of duplications (*duplication cost*), the number of losses (*loss cost*) or both combined (*mutation cost*). The most popular method for finding a parsimonious reconciliation is based on the LCA mapping between the gene tree and the species tree. The *LCA mapping *between *G *and *S*, denoted by *m*(), maps every node *x *of *G *to the LCA of L(x) in *S*. A node *x *of *G *is labeled as a duplication with respect to *S *if and only if *m*(*x_ℓ_*) = *m*(*x*) and/or *m*(*x_r_*) = *m*(*x*), where *x_ℓ _*is the left child of *x *and *x_r _*is the right child. Any node of *G *that is not a duplication node, is a speciation node. The LCA mapping induces a reconciliation *R *between *G *and *S*, where an internal node *x *of *G *leads to a duplication node in *R *if and only if *x *is a duplication node of *G *with respect to *S*. Moreover, *R *is a reconciliation that minimizes the duplication, loss, and mutation costs [24,26].

## Perspectives on homology

In the previous sections we vaguely referred to groups of genes from the same gene families as "homologous". In this section we solidify the notion of gene families by discussing the terminology related to homologous genes. There are many alternative definitions for homology and related concepts, the ambiguity being due to the many possible definitions of similarity between genes. Indeed, evolutionary, sequence, functional, or positional constraints give rise to definitions that are unfortunately not equivalent [27]. In this paper we adopt those definitions recommended by Fitch [23], corresponding to the evolutionary concepts.

**Definition 1 (homology) ***Two genes are *homologous *if and only if they descend, through duplication and speciation, from the same ancestral gene in the true evolutionary history*.

As the true history of genes is unknown, homology is usually inferred with some uncertainty, usually from amino acid or nucleotide similarity. Some confusion could remain about the definition of homology, since the belief is that all modern genes originated from a single gene or some small number of genes. In this context, all or most genes are homologous to all or most others. For this reason we posit that the evolutionary definition of homology might also include a notion of time. Fortunately, this issue does not have bearing on the results presented here.

The remainder of the definitions describe a hierarchy of homologous genes, implied by the true history of the genes.

**Definition 2 (orthology) ***Genes a and b are *orthologous *if and only if lca_H_*(*a*, *b*) *is a speciation node in the true history*.

As duplications may arise following a speciation event, the orthology relationship is not transitive. Thus it makes no sense to speak of sets/groups of orthologs. For example, in history *H *of Figure 1 the gene *a*_1 _is orthologous to the other four genes but *a*_2 _and *a*_3 _are not orthologous to *b*_2 _or *b*_3_. This property is inherent to the evolutionary definition of orthology, which is not a definition about the functional relationship between genes (Definition 3), nor the positional or direct descendant relationship that we introduce in more detail below (following Definition 3). Thus, the term *orthogroup *has been used to describe the set of genes that are orthologous according to a particular speciation [28]. Another distinction is the following.

**Definition 3 (isorthology) ***Two orthologous genes that have retained the same function as their LCA are called *isorthologous.

Isorthology has also been called *functional orthology*. In history *H *of Figure 1, the extant genes *a_i_*, for 1 ≤ *i *≤ 3 are pairwise isorthologous, which is not the case for any *a_i _*and *b_j_*. The isorthology relation is transitive. Therefore, it makes sense to speak of sets of isorthologs, or isorthogroups. Two genes are in the same *isorthogroup *if and only if they are isorthologous.

Notice that the notion of isorthology is different from that of "true exemplars" [29], "main orthologs" [30] or "positional homologs" [31] used in genome rearrangement studies, referring to direct descendants of the source gene of duplications, which are the ones likely to best reflect the original position of the ancestral genes in the ancestral genomes. Although the source gene is likely to preserve the parental function, this is not necessarily the case; the isorthology and direct orthology relationships are not equivalent.

**Definition 4 (paralogy) ***Genes a and b are *paralogous *if and only if lca_H_*(*a, b*) *is a duplication node in the true history*.

Duplications occur in an individual and copies can be fixed or lost on the population. From a functional point of view, the two copies (source and sink) of a duplication may evolve in different ways [32]: exactly one of the two copies preserves the parental function and the other copy either becomes non-functional (*pseudogenization*) or acquires a new function (*neofunctionalization*); both copies preserve the parental function (*bifunctionalization*) or collaborate in assuming a single function (*subfunctionalization*); or both copies lose function. From a sequence point of view, differentiation is likely to appear clearly in cases of pseudogenization or neofunctionalization. In these cases, the isorthology relationship is preserved, and the sequence distance between the parent gene and its isortholog descendant is likely to be shorter than its distance to the other copy, as the isortholog is constrained by function. In all other cases isorthology is not preserved, and the source and sink can not *a priori *be distinguished by comparison to the parental gene sequence or function. Notice also that only one of the source or sink assumes the isorthology relationship in the case of post-duplication evolution by pseudogenization or neofunctionalization, but not in case of the other post-duplication mechanisms.

## When reconciliation is not the right tool

The fundamental hypothesis behind reconciliation is that the gene tree reflects the true phylogeny of the gene family. Therefore, a strict prerequisite is to have both gene tree and species tree free from error. As demonstrated by many authors [7,8,33], few misplaced leaves in the gene tree induce a reconciliation with a significantly different mutation cost, and with a bias towards more ancient duplications (*i.e*. closer to the root of the history). In [24], internal nodes of the gene tree satisfying certain properties have been identified as problematic, and likely to be induced by misplaced leaves. Consequently, algorithms for correcting a gene tree prior to reconciliation have been developed [7].

In [8], it is stated that "most tree reconciliation analyses show biases, unless the gene trees used are exceptionally well-resolved and well-supported". As almost all gene trees are constructed from a multiple alignment of the gene sequences, well-supported gene trees require clear sequence differentiation. As detailed in the previous section, sequence differentiation is likely to arise following pseudofunctionalization or neofunctionalization. The case of subfunctionalization can lead to a variety of scenarios regarding sequence differentiation, bifunctionalization to sequence conservation, and loss of function to an excess of sequence differentiation eventually making the copies non-identifiable. Therefore, a set of genes that have evolved by pseudofunctionalization or neofunctionalization are the best data to use in reconciliation. We will assume that the following hypothesis holds.

### Hypothesis 1

*Following a duplication, exactly one of the source or sink genes preserve the parental function*.

For convenience, we coin the term *retainer *for the gene that retains the function after a duplication, and the term *mutant *for the gene that does not.

As pseudofunctionalization and neofunctionalization do not occur simultaneously with duplication, the underlying assumption in Hypothesis 1 is that enough time has passed after the duplication event to differentiate the two gene copies. Also notice that this hypothesis does not prevent subsequent functional loss. For example in the history *R *in Figure 1, a loss of the mutant arises after the duplication.

### Isolocalization

Errors in the gene tree are not the only reason for doubting reconciliation. A well-supported gene tree does not necessarily represent the true phylogeny for the gene family, as it is not necessarily the case that all genes have evolved at the same rate (*i.e*. with the same molecular clock). This is especially true in the case of post-duplication evolution by pseudofunctionalization or neofunctionalization, as isorthologs that are constrained by function are likely to evolve at a lower rate than the unconstrained copies [22]. This implies that in a well supported gene tree, the genes in an isorthogroup could appear as a subtree containing no other gene copies.

**Definition 5 (isolocalization property) ***A gene tree G respects the *isolocalization property *if and only if for any pair of isorthologous genes a*_1 _*and a*_2_*, the leaf-set $L\left(G_{x}\right)$ where x = lca_G_*(*a*_1_*, a*_2_)*, contains only genes that are isorthologous to a*_1 _*and a*_2_.

For example in Figure 1, according to the history *H*, the gene tree *G *respects the isolocalization property, whereas *P *does not.

The following theorem shows that reconciliation is not the correct tool for finding the true history when the isolocalization property holds. Say that a duplication node *d *in *H survives *if and only if there exist leaves *a *and *b *such that *lca_H_*(*a*, *b*) = *d*, and that a speciation node *s survives *if and only if there is a pair of isorthologs *a*_1_, *a*_2 _such that *lca_H_*(*a*_1_, *a*_2_) = *s*. For example, all speciation and duplication nodes in history *H *of Figure 1 are surviving nodes.

**Theorem 1 ***Take a history H with at least one surviving duplication node descending from one surviving speciation node. The version of H, with loss leaves removed, cannot be a gene tree that respects the isolocalization property*.

**Proof: **Let *G *be a gene tree that is a version of *H *with loss leaves (along with the internal node connecting the leaf to the tree) removed. Let *d *be a surviving duplication node that is a descendant of a surviving speciation node *s *in *G *(or similarly in *H*). Take a pair of isorthologs *a*_1_, *a*_2 _such that *lca_H _*(*a*_1_, *a*_2_) = *s*, and a gene *b *such that *lca_H _*(*a*_1_, *b*) = *d*. Thus, *b *is orthologous to, but not isorthologous to *a*_2_. Then *G *does not respect the isolocalization property, as the subtree of *G *rooted at node s contains, in addition to the two isorthologous genes *a*_1 _and *a*_2_, gene *b *which is not isorthologous to *a*_2 _   □

The following is the contrapositive of Theorem 1.

**Corollary 1 (isolocalization confounds reconciliation) ***A gene tree respecting the isolocalization property can never yield a reconciliation equivalent to the true history H with at least one surviving duplication node descending from one surviving speciation node*.

A more powerful but slightly more technical version of the corollary is the following. It highlights the fact that reconciliation could falter even when only part of the gene tree respects the isolocalization property.

**Corollary 2 ***A gene tree containing a subtree G' respecting the isolocalization property can never yield a reconciliation equivalent to a true history H such that: H has a speciation s with a pair of survivors *(*a*_1_*, a*_2_) *and a descendant duplication d with a pair of survivors *(*a*_2_, *b*), *where a*_1_, *a*_2 _*and b are leaves of G'*.

Figure 1 is the simplest example illustrating this negative result for reconciliation. If *H *were the true history, then the well-supported isolocalization respecting gene tree would be *G*. However, reconciliation of *G *with *S *leads to history *R*. This reconciliation erroneously places the duplication at the top of the tree and infers a loss which is absent from the true history. In this simple example, *G *and *P *lead to the same unrooted tree. However, according to Corollary 1 the problem is not always one of misrooting the gene tree, as a slightly larger history with an additional speciation or duplication event will continue to suffer from the same problem (as in the example from the last section).

## Isorthology respecting histories

Corollary 1 shows that reconciliation is not the right tool when a subtree of the gene tree adheres to the isolocalization property, yet there must be some information in the gene tree and species tree relationship. For instance, we expect subtrees corresponding to isorthologs in a well-supported gene tree to agree with the species tree. The following hypothesis formalizes this concept, where a tree *restricted *to a subset of leaves refers to the tree with all other leaves removed, and the edges around newly created single-child leaves contracted. In the rest of this paper, we will assume Hypothesis 2 in addition to Hypothesis 1.

### Hypothesis 2

*The gene tree G satisfies the isolocalization property and reflects the true phylogeny for the isorthogroups. Formally, for any isorthologous subset of genes *{*a_i_, a_j_, a_k_*}*, the tree G restricted to leaves a_i_, a_j_, and a_k_, but relabeled by s*(*a_i_*) *= i, s*(*a_j_*) *= j, and s*(*a_k_*) *= k, agrees with the species tree restricted to genomes i, j, and k*.

We now elaborate the connection between isorthologous genes and the LCA mapping. In what follows, nodes of *G *are labeled as duplication or speciation nodes according to the LCA mapping. The elementary proof of the following lemma is omitted due to space limitations.

**Lemma 1 ***Take a pair *(*a_i_, a_j_*) *of isorthologous genes, where a_i _is a gene in genome i, and a_j _is a gene in genome j. Then the node lca_G_*(*a_i_*, *a_j_*) *is a speciation node*.

Define a *speciation subtree *of *G *as a subtree such that all internal nodes (if any) are labeled as speciations by the LCA mapping. A corollary of Lemma 1 is that (under Hypothesis 2) isorthogroups of Γ are defined by speciation subtrees of *G*.

**Corollary 3 ***Any isorthogroup appears in G as the leaf-set of a speciation subtree*.

An *isorthologous subtree *of *G *is a speciation subtree of *G *corresponding to the leaves in an isorthogroup. Based on Corollary 3, the following definition introduces a natural alternative to reconciliation.

**Definition 6 (Isorthology Respecting History (IRH)) ***Given a gene tree G and a species tree S, a history H is an *isorthology respecting history *for *(*G*, *S*) *if and only if each isorthogroup induced by H is the leaf-set of a speciation subtree of G*.

In Figure 1, the histories *H *and *R *are isorthology respecting histories for the pair (*G*, *S*). Neither *H *nor *R *are isorthology respecting histories for the pair (*P*, *S*) since both histories imply isorthology between *a*_1 _and *a*_2_, but the pair does not.

Notice that Corollary 3 does not *a priori *give us the isorthogroups for pair (*G*, *S*), as the true isorthologous subtree could be part of a larger subtree of speciation nodes. For example, the left subtree of *G *in Figure 1 is consistent with three possible configurations of isorthogroups: {{*a*_1_}, {*a*_2_}, {*a*_3_}},{{*a*_1_}, {*a*_2_, *a*_3_}}, or {{*a*_1_, *a*_2_, *a*_3_}}. We will call an *isorthology respecting partition *of *G *a partition  P of L(G) such that each element of  P is the leaf set of a speciation subtree of *G*.

### Optimization problems

Following Corollary 3, an isorthologous respecting history appears as the most natural alternative to reconciliation. As many IRHs are possible for a given pair (*G*, *S*), an appropriate way for choosing most likely histories is required. Using a parsimony approach and either the duplication, lost, or mutation cost, the corresponding optimization problem is the following.

MINIMUM ISORTHOLOGY RESPECTING HISTORY

RECONSTRUCTION (MIRH):

**Input: **A gene tree *G *and species tree *S*.

**Output: **A *Minimum Isorthology Respecting History *(MIRH for short) for (*G*, *S*), *i.e*. an Isorthology Respecting History for (*G*, *S*) with minimum cost.

A restricted version of the MIRH problem would consider the maximal speciation subtrees of *G *as defining the isorthogroups. We later show that this isorthology respecting partition of *G *is the one that would minimize the duplication cost, but not necessarily the mutation cost.

The MIRH problem, as stated, ignores all the information on duplication and speciation nodes of *G *that are above the considered speciation subtrees. In other words, nothing is trusted in the gene tree except the isorthology information. An alternative would be to account for the hierarchy of the higher nodes in *G*.

**Definition 7 (Triplet Respecting History (TRH)) ***Let H be an isorthology respecting history for *(*G*, *S*)*, and P  be the isorthology respecting partition of G induced by H. Then H is a *triplet respecting history *if and only if for any triplet of genes *{*a, b, c*}*, where each gene is taken from a different isorthogroup of P , the tree G restricted to leaves a, b, and c agrees with H restricted to leaves a, b, and c*.

We can now formulate our second optimization problem.

MINIMUM TRIPLET RESPECTING HISTORY

RECONSTRUCTION (MTRH):

**Input: **A gene tree *G *and species tree *S*.

**Output: **A *Minimum Triplet Respecting History *(MTRH for short) for (*G*, *S*), *i.e*. a Triplet Respecting History for (*G*, *S*) of minimum cost.

Taking our model example in Figure 1, the true history *H *is a MIRH and a MTRH for the gene tree *G*, leading to a mutation and duplication cost of one (one duplication). Recall that *H *can never be recovered with a reconciliation when *G *respects the isolocalization property. Moreover the reconciliation *R *for *G *and *S *leads to a mutation cost that is higher (one duplication and one loss) than that of *H*. In this paper, we focus on the MIRH problem, which is the subject of the next section.

## On reconstructing isorthology respecting histories

In this section we justify the following algorithm to solve MIRH under the duplication cost. Start with a forest that corresponds to some isorthology respecting partition for (*G*, *S*). Then join the trees in a parsimonious way, using duplications and losses so as to respect the partition.

The groundwork for this approach has been established, where the implications of Hypothesis 2 linked isorthogroups with subtrees of speciation nodes in Corollary 3. The main result of this section is Theorem 2, and the supporting lemmas follow.

**Theorem 2 ***If  F is the set of n maximal speciation subtrees of G, then a *MIRH *for *(*G, S*) *will have duplication cost n *- 1.

**Proof: **Lemma 2 tells us that we need exactly *n *- 1 duplications for *n *isorthogroups. The minimum number of isorthogroups is implied by the set of maximal speciation subtrees of *G*.    □

A linear-time algorithm to compute the duplication cost of the MIRH will be given later.

### Inferring duplications

**Lemma 2 ***Take a *MIRH *H for *(*G*, *S*)*. H has n isorthogroups if and only if there are n - *1 *duplications in H*.

The rest of the subsection is a proof of Lemma 2.

**Proof: **We begin by proving some useful facts about the relationship between duplications and isorthologs. Say that  F is the set of isorthologous subtrees given by *H*.

**Lemma 3 (isorthologs by retainers) ***Consider some T∈ F and the path P in H between two leaves a,b∈L(T). For any duplication node d *∈ *P, a or b descends from the retainer of the duplication*.

**Proof: **Assume that *a *or *b *do not descend from the retainer of some duplication *d *∈ *P*. Then *a *and *b *are not isorthologous, a contradiction    □

**Lemma 4 (non-isorthologs by mutants) ***Consider the path P in H between leaves a *∈ *T*1 *and b *∈ *T*2 *such that T*1*, T2∈ F and T*1 *≠ T*2*. There exists some duplication node d *∈ *P such that either a or b descends from the mutant of d*.

Proof: Assume that there does not exist a duplication *d *such that *a *or *b *descend from the mutant of *d*. Then *a *and *b *are isorthologous, a contradiction    □

The following property follows directly from Lemmas 3 and 4.

**Property 1 ***Decompose the nodes of H into connected components *C *such that nodes a and b are in the same component if and only if a (resp. b) descends from the retainer of all duplications on the path from lca_H_*(*a*, *b*) *to a (resp. b). For each *C∈C, L(C)*is an isorthogroup*.

Since *H *is a parsimonious history, we get the following.

**Remark 1 ***For any duplication node d in a *MIRH *H, we have at least one gene in *Γ *that descends from the mutant of d*.

An implication of Property 1 is that the root of any isorthologous subtree must be joined to *H *by a duplication. Thus, a lower bound for the number of duplications in *H *is *n *- 1. But *n *- 1 is also an upper bound since, by Remark 1, more than *n *- 1 duplications implies more than *n *isorthogroups.

In the other direction, if there are *n *- 1 duplications then there must be at least *n *isorthogroups by Remark 1 and the fact that there is at least one gene in Γ from each genome in *S*. By Lemma 4, there are at most *n *isorthogroups in *H *   □

### The algorithm

We wrap up the section with a description of our algorithm that computes the duplication cost of the MIRH. Construction of the tree is straightforward, and is not presented here.

1. Label the nodes of *G *as speciations or duplications according to the LCA mapping.

2. Compute  F:

(a) Label all ancestors of a duplication node in a post-order traversal of *G*.

(b) Label each speciation node as a root of a tree in  F if its parent is a duplication or an ancestor of a duplication.

3. Return | F|-1.

The LCA mapping can be computed in linear time [34,35]. The two post-order traversals of *G *to compute  F can be done in linear time. Therefore, the total time to compute the duplication cost of a MIRH for (*G, S*) is linear in the size of *G*.

## Applications

**Figure 2:**
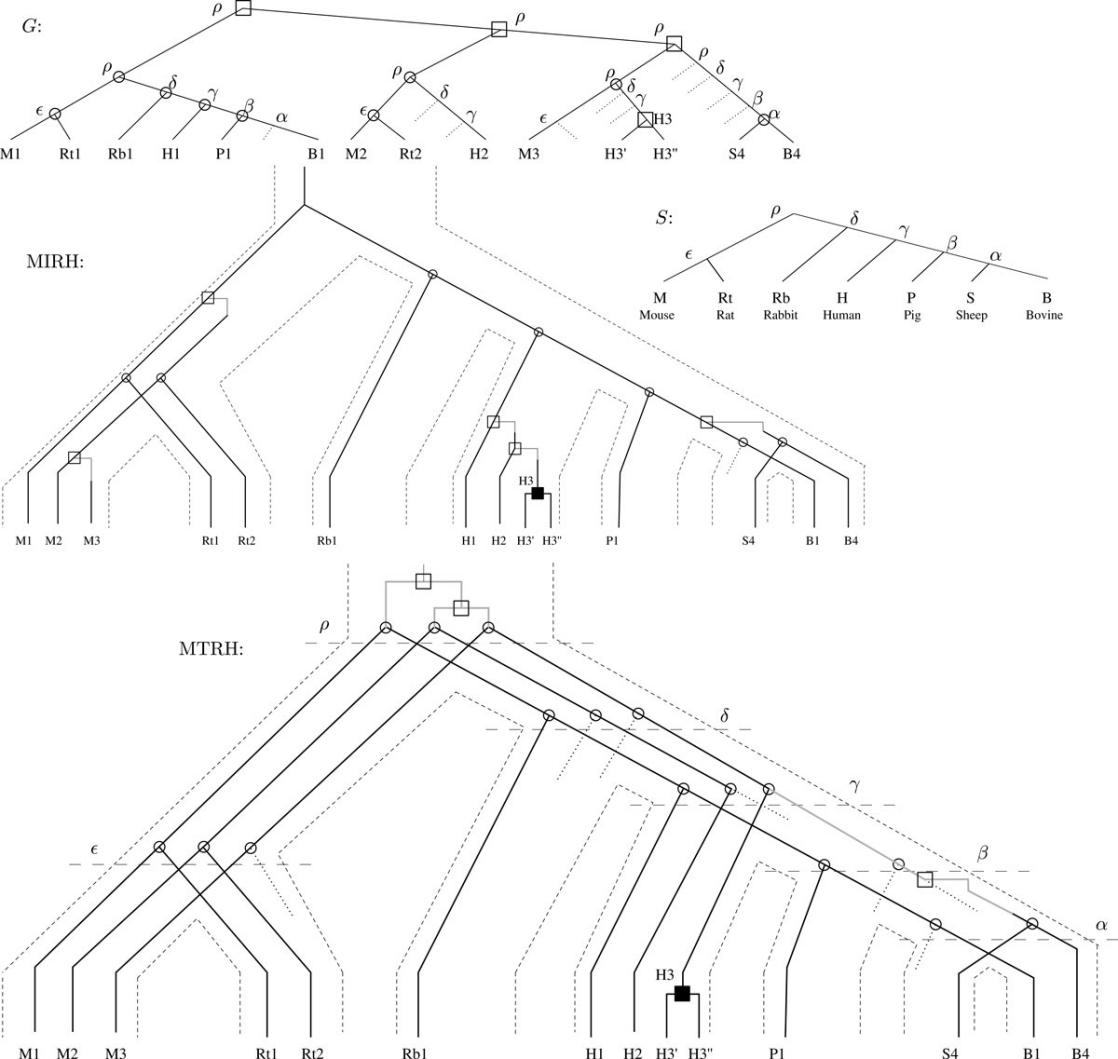
**Trees for mammals (*S*) and for mammalian interleukin-1 genes (solid lines in *G *) taken from **[22], **along with the reconciliation (the tree *G *augmented with the losses represented as dotted lines), the MTRH and the MIRH**.

A model example used by Page and Holmes [22] to illustrate the role of reconciliation is the mammalian interleukin-1 (IL) gene tree, represented in Figure 2(G). As there are four copies in human there must have been at least three duplications, while reconciling the interleukin tree with a tree for the mammals (Figure 2(S)) reveals an additional duplication. Ignore the recent duplication in the human lineage for a moment and consider H3' and H3" to be a single node H3. The three duplications are inferred at the root of the tree, which results in 10 losses (dotted lines in the gene tree *G*).

The reconciliation and MTRH lead to four isorthogroups ({*M*1, *Rt*1, *Rb*1, *H*1, *P*1, *B*1}, {*M*2, *Rt*2, *H*2}, {*M*3, *H*3}, {*S*4, *B*4}), whereas the MIRH leads to six (*H*2 is not considered isorthologous to {*M*2, *Rt*2}, and *H*3 is not isorthologous to *M*3). For the MTRH, three duplications (in addition to the recent duplication in the human lineage) are necessary to connect these trees. However, the duplication explaining the paralogy relationship between the X3 and X4 (where × is one of {M,Rt,Rb,H,P,S,B}) genes is now inferred to occur just before the most recent speciation event *α *leading to the Sheep and Bovine lineage. The reconciliation approach explains this paralogy relationship by a duplication at the root followed by four losses. Having a lower duplication decreases the mutation cost from 14 to 10 in the case of MTRH, but more importantly the mammalian ancestor for the interleukin gene family is now believed to have contained three copies of this gene instead of four. The less constrained MIRH infers a single ancestral gene copy and a mutation cost of 7.

In the MTRH of Figure 2 the gene M3 is interpreted as being isorthologous to H3 -- the source of the recent duplication leading to H3' and H3". It has to be noted however that this interpretation contradicts our strict definition of isorthogroups as corresponding to speciation subtrees. The hypothesis underlying this strict definition is that enough time has passed to functionally differentiate all products of duplications, and create isorthogroups (Hypothesis 2). Although this hypothesis is reasonable in the case of orthologous gene copies since speciation events can be assumed to be old enough, it is not appropriate for paralogous gene copies inside a genome that reflect strong sequence similarity. Preprocessing the gene tree by contracting each subtree that contains genes from only a single species is appropriate in this case.

## Discussion and future directions

Our theory is based on the assumption that enough time has passed to differentiate the products of each duplication (Hypothesis 2). In other words, even the most recent speciation events must be old enough to allow for clear formation of isorthogroups. Therefore, duplications that are inferred at the root by reconciliation will be lower in a MIRH. In terms of molecular clocks, this corresponds to an assumption of an infinite ratio between the molecular clock outside versus inside an isorthogroup. MTRH is more constrained in that it incorporates internal node information into the history. An alternative would be to allow for some constant ratio between rates. For example, an assumption that the substitution rate outside an isorthogroup is twice that of inside an isorthogroup would yield a different history. In the example of Figure 2, the duplication giving rise to the isorthogroup X4 could be situated at any level on the branch from *ρ *to *α*. Constraints on the molecular clocks would limit how far a duplication may "move". This paper formalizes the link between sequence similarity and function, and studies its effect on reconciliation. We bring to light the fact that under simple hypotheses, where a gene tree reconstruction is likely to yield an apparently good (statistically well-supported) gene tree, reconciliation does not lead to the true evolutionary history for most nontrivial cases. Hypothesis 1 assumes that a single gene copy preserves the parental function after a duplication, an assumption widely used by the community and confirmed experimentally in many cases [22]. The first part of Hypothesis 2 -- which states that the gene tree respects the isolocalization property -- is our attempt to formalize the link between sequence and function. This is an abstraction as we do not yet have a method to check for the existence of such a property on all or part of a gene tree, yet we expect that there are many gene trees in the literature or in public databases with at a least a subtree respecting the isolocalization property. Our main result on reconciliation still applies to a gene tree that does not respect the second part of Hypothesis 2 -- which states that the true phylogeny is reflected by the isorthogroups. However, such a gene tree would severely limit our ability to infer the correct history.

## Competing interests

The authors declare that they have no competing interests.
